# Developing a health education program for home enteral nutrition after esophageal cancer surgery based on the Delphi method

**DOI:** 10.1097/MD.0000000000041586

**Published:** 2025-02-21

**Authors:** Weiran Tian, Wei Liu, Cuirong Wang, Lei Liu, Liming Zhang, Lu Chen

**Affiliations:** a Department of Thoracic Surgery, The Fourth Hospital of Hebei Medical University, Shijiazhuang, Hebei, China; b Department of Breast Surgery, The Fourth Hospital of Shijiazhuang, Shijiazhuang, Hebei, China; c Department of Otolaryngology, The Fourth Hospital of Hebei Medical University, Shijiazhuang, Hebei, China; d Department of Breast Surgery, The Fourth Hospital of Hebei Medical University, Shijiazhuang, Hebei, China; e Department of Nursing, The Fourth Hospital of Hebei Medical University, Shijiazhuang, Hebei, China.

**Keywords:** Delphi method, esophageal cancer, health education, home enteral nutrition, nursing

## Abstract

To develop a health education program for home enteral nutrition (HEN) after Esophageal cancer (EC) surgery based on Delphi method, providing reference opinions for clinical nursing education and patient home care. The health education program for HEN after EC surgery was constructed through literature research, theoretical analysis and group discussion. From February to June 2024, experts in clinical nursing, clinical medicine, and nutrition of EC were invited to conduct 2 rounds of Delphi expert consultation to initially determine the items of the program. The weight and assignment of each items were determined through analytic hierarchy process (AHP), and then the final health education program for HEN after EC surgery was established. The authoritative coefficients of the 2 rounds consulting experts were 0.918 and 0.929 and the positive coefficients were 100% and 95%, respectively; The mean range of importance assignment for the second round of consultation indicators was 4.26 to 5.00 points, with a full score range of 40.90% to 95.45%. The mean harmony coefficients for expert opinions from 2 rounds were 0.206 and 0.218 (*P* < .01), respectively. The final health education program for postoperative HEN in EC includes 6 primary items, 27 secondary items, and 18 tertiary items. The results of the AHP showed that the consistency coefficients (CR values) of each matrix for the tertiary items were all < 0.1, meeting the requirements of consistency testing. The constructed health education program for postoperative HEN patients with EC in this study has high scientific and practical value, and can provide reference for the health education of postoperative HEN patients with EC.

## 1. Introduction

Esophageal cancer (EC) is a worldwide health issue and an invasive malignant tumor.^[1]^ Surgery is currently the preferred treatment for EC.^[2]^ With the increasing acceptance of the enhanced recovery after surgery concept by clinicians, the patients’ postoperative hospitalization time have been shortened and they cannot fully recover from oral intake of sufficient energy upon discharge.^[3]^ Providing home enteral nutrition (HEN) for postoperative patients can extend nutritional support from hospital to home, enabling patients to receive long-term and effective nutritional support treatment outside the hospital.^[4,5]^

Patients with EC frequently suffer from dysphagia, which, exacerbated by the depletion caused by the tumor, leads to nutritional deficiencies. Dysphagia and surgical trauma further contribute to a significant decline in postoperative nutrition, immune function, and quality of life.^[6]^ Thoracic surgeons primarily rely on enteral nutrition as a nutritional support method. Previous research has indicated that early postoperative total enteral nutrition for high-risk surgical patients can effectively decrease the incidence of postoperative complications.^[7]^ Adequate training provided by qualified professionals to patients and/or their families is a necessary condition for the feasibility of HEN. In terms of patient monitoring and follow-up, coordination among multidisciplinary teams is crucial for preventing complications such as malnutrition and dehydration in these patients, as well as complications caused by enteral nutrition itself, such as vomiting, diarrhea, constipation, and abdominal distension.^[8]^ However, some studies have reported that 25% to 35% of patients experience mechanical complications due to HEN,^[9]^ and some patients experienced gastrointestinal discomfort symptoms,^[10]^ only a small number of caregivers know how to prevent and treat HEN complications, and only 28.57% of caregivers are proficient in enteral nutrition pipeline care.^[11]^ It is evident that there is no systematic management guidance system for HEN, and the relevant professional knowledge of HEN patients and caregivers is relatively lacking. To improve the nursing ability of caregivers and ensure the safety and effectiveness of HEN after EC surgery, this study constructed a health education program for HEN based on the Delphi method, providing theoretical guidance for the health education of postoperative HEN patients with EC.

## 2. Materials and methods

### 2.1. Establishment of the research group

The research group consists of 7 members, all with over 10 years of experience in medical nursing for EC, including 2 thoracic surgeons and 5 nurses; The group members are tasked with project design, developing expert consultation questionnaires, ensuring research quality control, and selecting consulting experts. Two nurses are responsible for literature retrieval, questionnaire distribution and collection, as well as summarizing and analyzing expert opinions. This study was approved by the Ethics Committee of the Fourth Hospital of Hebei Medical University (2023KS207).

### 2.2. Forming preliminary draft of postoperative HEN health education plan for EC

The research group searched Pub Med, Web of Science, Embase, Cochrane library, and other databases. The search term is “esophageal neoplams,” “esophagus cancer,” “enteral nutrition,” “tube feeding,” “gastric feeding tubes,” “health education,” “community health education,” and “health promotion.” The search deadline is from database establishment to April 2024. Delete the literature that cannot obtain the full text, has duplicate content, and low quality. Ultimately, 14 articles and 3 books were selected, including 7 randomized controlled trials, 4 guidelines, and 6 systematic reviews. The members of the research group analyzed the included literature and referred to the current health education program for HEN after EC surgery. After group discussion, a preliminary draft of the health education program for HEN after EC surgery was formed, which included 6 primary items, 27 secondary items, and 18 tertiary items.

### 2.3. Development of expert consultation questionnaire

The expert consultation questionnaire was developed based on the preliminary draft of the patient’s health education program, which includes 3 sections: questionnaire introduction: detailing the significance, background, purpose of the consultation, and instructions for completing the questionnaire. Expert information: including basic information about the experts, familiarity with the questionnaire content. Questionnaire subjects: uses Likert 5-level scoring method,^[12]^ experts evaluate the importance and feasibility of each item in the primary, secondary, and tertiary health education items for HEN after EC surgery. In addition, in order to facilitate experts to fully express their opinions on modifying and deleting items, each item is accompanied by a column for comments on modification and supplement.

### 2.4. Selection of consulting experts

Using purposive sampling method,^[13]^ we selected representative experts from 7 different provinces in China from the expert database of the Chinese Medical Association. The research areas of these senior experts include clinical thoracic surgery, nutrition, and nursing. After group discussion, a total of 22 experts were selected. To ensure the authority of the experts, the inclusion criteria for experts in this study were: bachelor degree or above; more than 10 years of work experience, with rich theoretical knowledge and practical experience in the treatment and nursing of EC; experts are highly motivated and volunteer to participate in this study.

### 2.5. Implementation of expert consultation

This study conducted 2 rounds of expert consultation, utilizing a combination of paper and electronic questionnaires, and required experts to respond within 1 week. The interval between 2 rounds of consultation is 3 to 4 weeks. Summarize the scoring of the first round of expert consultation, following the principles of importance assignment mean score ≥ 4.0 points, coefficient of variation ≤ 0.25, and full score ratio > 0.20.^[13]^ Combining expert opinions and revision suggestions, the entries were modified through data verification and group discussion, forming the second round of expert consultation form. After the second round of expert consultation, the results were analyzed and the entries at all levels were revised based on expert suggestions. After 2 rounds of consultation, the expert opinions were essentially unanimous, and the consultation was concluded.

### 2.6. Statistical analysis

Data were entered and analyzed by 2 people using Excel and SPSS 27.0 (Chicago). The importance scores of items at all levels were expressed as mean ± standard deviation (x ± s). The level of expert enthusiasm was expressed by the questionnaire recovery rate and the ratio of experts who provided suggestions. The questionnaire recovery rate ≥ 70% indicates that the enthusiasm of experts is good.^[14]^ The degree of expert authority is represented by the authority coefficient (Cr), which is determined by the expert’s judgment basis coefficient (Ca) and the expert’s familiarity coefficient (Cs) with the item. Cr = (Ca + Cs)/2, Cr > 0.80 indicates a high level of authority among experts.^[15,16]^ The level of consensus among expert opinions is represented by the coefficient of variation and the Kendall harmony coefficient (W). The analytic hierarchy process (AHP) is employed to develop a judgment matrix, and the weight vector for the indicators is determined by assessing the significance of the matrix elements. The weights for primary, secondary, and tertiary items are then calculated. By establishing a judgment matrix, a hierarchical single ranking and consistency test are conducted to determine the weights and combined weights of each indicator, then a consistency coefficient (CR value) was obtained. If CR < 0.1, it can be considered that the construction of the judgment matrix is reasonable,^[16]^ with *P* < .05 considered statistically significant.

## 3. Results

### 3.1. General information of experts

This study conducted 2 rounds of expert consultation, and ultimately selected 22 experts from regions including Hebei, Henan, Hunan, Beijing, Tianjin, Jiangsu, Shanghai in China. These experts span the fields of thoracic surgery, nutrition, and clinical nursing. Additional general information is provided in Table 1.

**Table 1:** General information of experts (n = 22).

Project		Number	Ratio
Gender	Male	6	0.273
	Female	16	0.727
Education	Undergraduate	8	0.364
	Master	11	0.500
	Doctor	3	0.136
Year of work experience	10 to 20 yr	7	0.318
	21 to 30 yr	6	0.273
	Over 30 yr	9	0.409
Specialized field	Clinical medicine in thoracic surgery	4	0.182
	Nursing in thoracic surgery	16	0.727
	Nutrition	2	0.091

### 3.2. Expert activity level

In the first round of expert consultation, 22 questionnaires were distributed and 22 were collected, achieving a 100% effective response rate. In the second round, 22 questionnaires were distributed and 21 were collected, with an effective response rate of 95.5%. This demonstrates a high level of enthusiasm among the experts for participating in this study. The number of experts suggesting modifications in the 2 rounds of consultation was 14 (63.6%) and 2 (9.1%), respectively, indicating a convergence in expert opinions.

### 3.3. Degree of expert authority

The degree of expert authority is represented by the authority coefficient (Cr). Cr = (Ca + Cs)/2, Cr > 0.80 indicates a high level of authority among experts. The expert’s judgment basis coefficient (Ca) for the scale content encompasses 4 components: theoretical basis, practical experience, whether to consult relevant materials, and intuitive perception. It is evaluated using 3 levels: high, medium, and low. Theoretical basis is assigned values of 0.3, 0.2, and 0.1; practical experience, 0.5, 0.4, and 0.3; consult relevant materials, 0.1, 0.1, and 0.1; and intuitive judgment, 0.1, 0.1, and 0.1. The expert’s familiarity coefficient with the scale content (Cs) is categorized into 5 levels: very familiar (1.0), relatively familiar (0.8), general (0.5), not very familiar (0.2), and unfamiliar (0). The Ca values for the 2 rounds of consultations are 0.950 and 0.952, respectively, and the Cs are 0.886 and 0.905, respectively. Cr = (Ca + Cs)/2. The authority coefficients for both rounds of expert consultations are 0.918 and 0.929, both > 0.8 (Table 2). Therefore, it can be concluded that the expertise in this study is highly authoritative, and the results of the consultations are credible.

**Table 2:** Expert authority degrees of health education programs.

	Number	Ca	Cs	Cr
First round	22	0.950	0.886	0.918
Second round	21	0.952	0.905	0.929

### 3.4. The centralization and harmonization among expert opinions

The centralization degree of expert opinions is represented by the importance score and coefficient of variation. The importance score and coefficient of variation of the 2 rounds expert consultation meet the items screening criteria, and the significance test of the harmony coefficient is *P* < .01 (Table 3).

**Table 3:** The degree of centralization and harmonization of the 2 rounds expert opinions.

Rounds	Importance score	Coefficient of variation	Kendall harmony coefficient	χ^2^	*P*
First round	4.85 ± 0.39	0.00 to 0.20	0.254	69.424	<.001
Second round	4.78 ± 0.42	0.00 to 0.21	0.248	76.556	<.001

### 3.5. Expert consultation results

The first round of expert consultation questionnaire included 6 primary items, 27 secondary items, and 18 tertiary items. Based on the experts’ suggestions and the statistical requirements of the Delphi method, the items were revised as follows: delete 3 tertiary items that have an average importance score of <4.0 points and a coefficient of variation exceeding 0.25 points, including “precautions for nitrogen balance monitoring,” “prevention of infection at the stoma,” and “adjustment of infusion based on scoring results.” revised 8 secondary and tertiary items: for example, “the method of stopping HEN” was changed to “Extubation method for termination of HEN,” “calculating daily nutritional requirements” was changed to “the daily nutrient requirements for patients,” “risk assessment before rehabilitation exercise” was revised to “exercise contraindications and termination indicators,” and “the manifestations of enteral nutrition intolerance” and “the simple assessment of enteral nutrition tolerance” were changed to “prevention and treatment of nausea and vomiting,” “prevention and treatment of abdominal distension,” “prevention and treatment of diarrhea,” “prevention and treatment of constipation,” etc. Split 2 tertiary items: “monitoring precautions for weight and urine volume” into “monitoring precautions for weight” and “monitoring precautions for urine volume,” and “prevention and treatment of catheter blockage and dislocation” into “prevention and treatment of catheter blockage” and “prevention and treatment of catheter displacement.” Add 5 secondary and tertiary items: “approaches to HEN,” “intermittent Bolus injection method,” “changes in digestive tract structure and function following EC surgery,” “methods for transitioning to a normal diet.” After the second round of consultations, no items were modified, and the final educational program constructed included 6 primary items, 27 secondary items, and 18 tertiary items. Calculate the weight vector of indicators based on the importance of matrix elements, and determine the weights of primary, secondary, and tertiary items. Combining the results of experts consultation with the AHP, the weights and combined weights of each item were determined, and the CR values of all items were <0.1 (Table 4).

**Table 4:** Expert consultation outcomes on developing a health education program for postoperative HEN in esophageal cancer patients based on the Delphi method.

Items	Importance score (x ± s)	Coefficient of variation	Weight	Combined weight	Maximum characteristic root	CR value
Ⅰ Knowledge about HEN	4.71 ± 0.46	0.098	0.0656	0.0656	6.0092	0.002
Ⅰ-1 The meaning and advantages of HEN	4.33 ± 0.66	0.152	0.0365	0.0090	3.0183	0.018
Ⅰ-2 Types and selection of enteral nutrition preparations	4.43 ± 0.68	0.153	0.2385	0.0156		
Ⅰ-3 Storage method of enteral nutrition preparation	4.86 ± 0.48	0.098	0.6250	0.0410		
Ⅱ Application method of HEN	5.00 ± 0.00	0	0.2065	0.2065	6.0092	0.002
Ⅱ-1 Approaches to home enteral nutrition	4.52 ± 0.75	0.166	0.0822	0.0170	6.1130	0.018
Ⅱ-2 Method of home tube feeding enteral nutrition infusion	4.67 ± 0.66	0.141	0.1186	0.0245		
Ⅱ-2-1 Continuous/intermittent infusion method with nutrition pump	4.76 ± 0.54	0.113	0.5278	0.0129	3.0536	0.052
Ⅱ-2-2 Continuous/intermittent infusion by gravity	4.29 ± 0.96	0.223	0.1396	0.0034		
Ⅱ-2-3 Intermittent Bolus injection method	4.26 ± 0.59	0.128	0.3325	0.0081		
Ⅱ-3 Precautions for HEN infusion	4.90 ± 0.44	0.089	0.2054	0.0424	6.1130	0.018
Ⅱ-4 Fixation and maintenance of home enteral nutrition pipeline	5.00 ± 0.00	0.000	0.3261	0.0673		
Ⅱ-5 Extubation method for termination of home enteral nutrition	4.48 ± 0.68	0.152	0.0622	0.0128		
Ⅱ-6 Precautions for using the enteral nutrition pump	4.90 ± 0.30	0.061	0.2054	0.0424		
Ⅱ-6-1 Routine maintenance of enteral nutrition pump	4.67 ± 0.48	0.104	0.5000	0.0212	2.0000	<0.001
Ⅱ-6-2 Alarm response procedures for the enteral nutrition pump	4.67 ± 0.51	0.110	0.5000	0.0212		
Ⅲ Monitoring the effect of home enteral nutrition	5.00 ± 0.00	0.000	0.2065	0.2065	6.0092	0.002
Ⅲ-1 Monitoring Indicators for HEN	4.67 ± 0.48	0.104	0.2970	0.0613	3.0092	0.009
Ⅲ-2 Significance of home enteral nutrition monitoring	4.48 ± 0.60	0.134	0.1634	0.0337		
Ⅲ-3 Monitoring and precautions for home enteral nutrition	4.76 ± 0.54	0.113	0.5396	0.1114		
Ⅲ-3-1 Monitoring precautions for weight	4.76 ± 0.44	0.092	0.5396	0.0601	3.0092	0.009
Ⅲ-3-2 Monitoring precautions for urine volume	4.52 ± 0.60	0.133	0.2970	0.0339		
Ⅲ-3-3 Biochemical monitoring and precautions	4.43 ± 0.60	0.135	0.1634	0.0182		
IV Prevention and management of complications	5.00 ± 0.00	0.000	0.2065	0.2065	6.0092	0.002
IV-1 Management of gastrointestinal complications	4.95 ± 0.22	0.044	0.4133	0.0853	4.0709	0.027
IV-1-1 Prevention and treatment of nausea and vomiting	4.95 ± 0.22	0.044	0.1429	0.0122	4.0000	<0.001
IV-1-2 Prevention and treatment of abdominal distension	5.00 ± 0.00	0.000	0.2857	0.0244		
IV-1-3 Prevention and treatment of diarrhea	5.00 ± 0.00	0.000	0.2857	0.0244		
IV-1-4 Prevention and treatment of constipation	5.00 ± 0.00	0.000	0.2857	0.0244		
IV-2 Management of mechanical complications	4.81 ± 0.51	0.106	0.1867	0.0386	4.0709	0.027
IV-2-1 Prevention and treatment of catheter blockage	4.86 ± 0.48	0.098	0.5000	0.0193	2.0000	<0.001
IV-2-2 Prevention and treatment of catheter displacement	4.86 ± 0.48	0.098	0.5000	0.0193		
IV-3 Management of infectious complications	4.86 ± 0.36	0.074	0.2922	0.0603	4.0709	0.027
IV-3-1 Preventive measures for aspiration pneumonia	5.00 ± 0.00	0.000	0.7500	0.0453	2.000	<0.001
IV-3-2 Preventive measures for stoma infection	4.67 ± 0.58	0.124	0.2500	0.0151		
IV-4 Management of metabolic complications	4.57 ± 0.68	0.148	0.1078	0.0223	4.0709	0.027
IV-4-1 Recognition and treatment of hyperglycemia and hypoglycemia symptoms	4.90 ± 0.30	0.061	0.7500	0.0167	2.0000	<0.001
IV-4-2 Identification and treatment of low potassium and low sodium symptoms	4.57 ± 0.60	0.131	0.2500	0.0056		
V Methods for Transitioning to a Normal Diet	5.00 ± 0.00	0.000	0.2065	0.2065	6.0092	0.002
V-1 Changes in digestive tract structure and function following esophageal cancer surgery	4.57 ± 0.68	0.148	0.0577	0.0119	7.1496	0.018
V-2 The appropriate time for patient’s oral intake	4.9 ± 0.44	0.089	0.1878	0.0388		
V-3 The daily nutrient requirements for patients	4.62 ± 0.59	0.128	0.0703	0.0145		
V-4 Assessment method of swallowing function	4.67 ± 0.48	0.104	0.1062	0.0219		
V-5 Classification of transitional diets	4.67 ± 0.48	0.104	0.1062	0.0219		
V-6 Methods for transitioning to a normal diet	4.90 ± 0.30	0.061	0.1878	0.0388		
V-7 Precautions of transitional diets	4.95 ± 0.22	0.044	0.2839	0.0586		
Ⅵ Rehabilitation exercise during HEN	4.76 ± 0.44	0.092	0.1083	0.1083	6.0092	0.002
Ⅵ-1 Purpose of rehabilitation exercise	4.48 ± 0.60	0.134	0.0975	0.0106	4.0604	0.023
Ⅵ-2 Selection of exercise mode	4.76 ± 0.44	0.092	0.2068	0.0224		
Ⅵ-3 Exercise contraindications and termination indicators	4.90 ± 0.44	0.089	0.3478	0.0377		
Ⅵ-4 Precautions during rehabilitation exercise	4.90 ± 0.30	0.061	0.3478	0.0377		

Abbreviation: HEN = home enteral nutrition.

## 4. Discussion

Patients undergoing EC surgery exhibit a diminished capacity in self-care and a reduced quality of life, which is attributed to the digestive tract reconstruction and the substantial surgical trauma.^[17,18]^ After surgery, patients must adopt a new dietary pattern and it takes approximately 1 month to gradually transition to a soft diet. Consequently, it is essential to provide necessary nursing knowledge and skills for patients with EC undergoing HEN and their caregivers, so as to improve their nursing ability and subsequently elevating the life quality of patients.^[19,20]^

Based on a comprehensive literature review on EC, enteral nutrition, health education, this study initially developed a draft of a health education program for HEN after esophagectomy through group discussions. After 2 rounds of Delphi method implementation, combined with items screening criteria and expert advice, and through hierarchical analysis, a final health education program for HEN after esophagectomy was developed. The 22 consulting experts selected for this study came from 10 hospitals across 7 regions of China, taking into full consideration their professional fields and regional distribution. The selected experts are highly experienced, and 15 of them have more than 20 years of work experience; 14 of them have master’s degrees or higher. Their work spans across multiple disciplines, including medicine, nursing, and nutrition. The recovery rates of the 2 rounds of consultation questionnaires in this study were 100% and 95.5%, respectively. The proportion of experts who proposed modification suggestions in the 2 rounds was 63.6% and 9.1%, respectively, indicating that experts had a highly attention and enthusiasm for this study. According to the reference,^[15]^ Cr > 0.7 indicates that the expert possesses authority, while Cr > 0.8 signifies high authority. In this study, the Cr of the experts in 2 rounds was 0.918 and 0.929, respectively, indicating that the experts had high reliability and authority. The Kendall harmony coefficients of the 2 rounds were 0.254 and 0.248, respectively (*P* < .01), suggesting that the experts’ opinions were relatively consistent.

In addition, this study integrates the Delphi method with the AHP to assess the consistency of the weights of each item. The CR value of each level item is <0.1, demonstrating that the weight distribution within the item system is reasonable. Therefore, the postoperative HEN health education program for EC constructed in this study has significant reliability and scientific validity. The HEN health education program for postoperative patients with EC constructed by Delphi method in this study can provide scientific and reasonable health education guidance for the patients, which is conducive to the effective, comprehensive and standardized evaluation of health education for clinical nurses. It facilitates clinical nurses and caregivers in efficiently, comprehensively, and normatively assessing the effectiveness of health education. However, subjective factors from experts may have a certain degree of bias on the research results. Therefore, the feasibility and effectiveness of health education programs still need to be further verified in future clinical practice.

## 5. Conclusions

In conclusion, we developed a health education program for HEN after EC surgery based on Delphi method. The program was constructed through literature research, theoretical analysis, expert consultation and group discussion. Following 2 rounds of Delphi expert consultation, the final health education program for postoperative HEN in EC includes 6 primary items, 27 secondary items, and 18 tertiary items. The constructed health education program in this study has high scientific and practical value, and can provide reference for the health education of postoperative HEN patients with EC.

## Author contributions

**Conceptualization:** Lu Chen.

**Data curation:** Weiran Tian, Wei Liu.

**Formal analysis:** Weiran Tian, Lei Liu, Liming Zhang.

**Investigation:** Cuirong Wang.

**Methodology:** Weiran Tian, Cuirong Wang, Lei Liu, Liming Zhang.

**Writing – original draft:** Weiran Tian, Wei Liu.

**Writing – review & editing:** Lu Chen.
